# The circFASN/miR-33a pathway participates in tacrolimus-induced dysregulation of hepatic triglyceride homeostasis

**DOI:** 10.1038/s41392-020-0105-2

**Published:** 2020-03-27

**Authors:** Chenzhi Zhang, Kangchen Chen, Rongli Wei, Guanghan Fan, Xuechun Cai, Li Xu, Beini Cen, Jianguo Wang, Haiyang Xie, Shusen Zheng, Xiao Xu

**Affiliations:** 1 Division of Hepatobiliary and Pancreatic Surgery, Department of Surgery, First Affiliated Hospital, Zhejiang University School of Medicine, Hangzhou, 310000 China; 2 Key Lab of Combined Multi-Organ Transplantation, Ministry of Public Health, Hangzhou, 310000 China; 3 Department of Hepatobiliary and Pancreatic Surgery, Shulan (Hangzhou) Hospital, Hangzhou, 310000 China

**Keywords:** Metabolic disorders, Non-coding RNAs

## Abstract

Dyslipidemia exhibits a high incidence after liver transplantation, in which tacrolimus, a widely used immunosuppressant, plays a fundamental role. MicroRNAs and related circRNAs represent a class of noncoding RNAs that have been recognized as important regulators of genes associated with lipid metabolism. However, their transcriptional activities and functional mechanisms in tacrolimus-related dyslipidemia remain unclear. In this study, we observed that tacrolimus could induce triglyceride accumulation in hepatocytes by stimulating sterol response element-binding proteins (SREBPs) and miR-33a. Our in silico and experimental analyses identified miR-33a as a direct target of circFASN. Tacrolimus could downregulate circFASN and result in elevated miR-33a in vivo and in vitro. Overexpression of circFASN or silencing of miR-33a decreased the promoting effects of tacrolimus on triglyceride accumulation. Clinically, the incidence of dyslipidemia in liver transplant recipients with elevated serum miR-33a after liver transplantation was higher than that in patients without elevated serum miR-33a (46.3% vs. 18.8% *p* = 0.012, *n* = 73). Our results showed that the circFASN/miR-33a regulatory system plays a distinct role in tacrolimus-induced disruption of lipid homeostasis. MiR-33a is likely a risk factor for tacrolimus-related dyslipidemia, providing a potential therapeutic target to combat tacrolimus-induced dyslipidemia after liver transplantation.

## Introduction

Liver transplantation (LT) is widely accepted as the most efficient therapy for all forms of end-stage liver disease. However, recipients of liver transplants are at high risk of developing metabolic syndromes (MS), which seriously affect the long-term efficacy of liver transplants.^1^ MS have a prevalence of 44–58%^1–3^ following LT, and dyslipidemia, a prominent clinical manifestation of MS,^4,5^ has a prevalence of up to 70% in recipients of liver transplants.^1,2,6^ Immunosuppressive medications, particularly calcineurin inhibitors (CNIs), such as cyclosporine A (CsA) and tacrolimus (TAC), play a role in the development of dyslipidemia after LT.^7–9^ Several mechanisms have been reported for impaired lipid metabolism due to CNIs, such as LDL particle internalization,^10^ which causes an increase in LDL-C, reduction in hepatic triglyceride lipase,^11^ inhibition of bile acid synthesis from cholesterol,^12^ and increased susceptibility of LDL to oxidation.^13^ However, the mechanisms underlying hyperlipidemia in patients after transplantation and post-administration of CNIs are unclear. TAC is currently more widely used than CsA as an immunosuppressant after liver transplantation, and there is growing evidence that TAC could disrupt lipid metabolism homeostasis after LT. We, therefore, aimed to elucidate the role of TAC in lipid metabolism impairment.

MicroRNAs (miRNAs, miRs) are ~22-nt-long natural noncoding sequences that control the expression of genes associated with numerous pathological processes, including hepatic steatosis and dyslipidemia.^14–16^ MiR-33 is a typical miRNA; Horie et al. showed that miR-33 KO mice developed obesity and had elevated circulating triacylglycerol (TG) over time (after ~1 year in chow-fed mice or after 12 weeks in mice on a high-fat diet, HFD).^17^ Rayner et al. found that miR-33 contributes to the regulation of lipid homeostasis through ABCA1 and ABCG1; overexpression of miR-33 in mouse liver resulted in a decrease in ABCA1 and ABCG1 expression and a corresponding decrease in plasma HDL-C levels. The miR-33 family comprises intronic miRNAs encoded within the sterol response element-binding protein (SREBP) genes.^18,19^ SREBPs are transcription factors that bind to the sterol regulatory element and the master transcriptional regulators of lipid and sterol biosynthesis. SREBPs consist of three isoforms: 1a, 1c, and 2. SREBP-1a and SREBP-2 are involved in hepatic lipogenesis and activate fatty acid and cholesterol synthesis via the PI3K/AKT pathway.^20,21^ Since TAC reduces AKT protein phosphorylation, we speculated that TAC could influence the function of miR-33 and SREBPs.

Not every recipient of a liver transplant under TAC treatment develops dyslipidemia, indicating a likely feedback regulatory system against the influence of TAC on miR-33. Because noncoding RNAs, such as circular RNAs (circRNAs) regulate miRNA function, we speculated that circRNAs might be involved in the regulation of miR-33 function and feedback regulation. CircRNAs are a class of noncoding RNAs with connected 3ʹ and 5ʹ ends, which are formed by back-splicing events through exon or intron circularization.^22^ Several circRNAs play important roles in biological processes by “sponging” target miRNAs and regulating the expression of miRNA-targeted transcripts.^23^ We hypothesized that TAC might disrupt lipid metabolism homeostasis through a circRNA-miRNA-mRNA pathway and thereby induce dyslipidemia after liver transplantation. In this study, we aimed to identify the circRNA, if any, that regulates lipid metabolism by “sponging” miR-33 and acts as a feedback regulator to maintain lipid metabolism homeostasis after LT. We also aimed to elucidate the effects of TAC on lipid metabolism and dyslipidemia after liver transplantation.

## Materials and methods

### Animal experiments

Male C57BL/6 mice (age: 4 weeks, body weight: 18–20 g) were obtained from Zhejiang Academy of Medical Sciences and housed in cages at 20 °C under a 12:12 h light–dark cycle. Mice were fed a normal chow diet. Animal experiments were approved by the Institutional Animal Care and Use Committee and the Ethics Committee of Zhejiang University.

Twenty-four mice were randomly allocated into three treatment groups. Mice were intraperitoneally injected daily in the morning with saline (control group) or TAC (0.5 or 2.0 mg/kg/day), which was supplied by Selleck Chemicals LLC (Houston, TX, USA). Body weight was regularly recorded every week. Animals were sacrificed 8 weeks after the initial TAC injection. Peripheral blood was extracted for biochemical analysis, including serum TC and TG; liver tissues were removed to detect triglyceride amounts by oil red O staining, miR-33a and circFASN expression by RT-PCR, and relative protein expression by western blot.

### Cell culture and transfection

Hep-G2 and Huh7 (human hepatocellular adenocarcinoma) cell lines were purchased from the Cell Bank of the Chinese Academy of Sciences (Shanghai, China). Hep-G2 and Huh7 cells were maintained in DMEM (Gibco, USA) supplemented with 10% FBS at 37 °C in a humidified atmosphere of 95% air with 5% CO_2_.

For circFASN overexpression and silencing, the circFASN plasmid and siRNA targeting the junction region of circFASN were purchased from Guangzhou Geneseed Biotech Co (Guangzhou, China). Hep-G2 and Huh7 cells were transfected with the overexpression plasmid or siRNA by using Lipo3000 Transfection Reagent (Invitrogen, USA).

For miR-33a overexpression and inhibition, Hep-G2 and Huh7 cells were transfected with 50 nM miRNA mimics (miR-33a) or 100 nM miRNA inhibitor (anti-miR-33a) (GenePharma, Shanghai, China) by using Lipo3000 Transfection Reagent (Invitrogen). Control groups were treated with equal concentrations of nontargeting mimic or inhibitor negative control sequences to control for nonspecific effects.

### Bioinformatics

To determine the upstream circRNAs of miR-33a, three different algorithms, circBase (http://www.circbase.org/), starBase (http://www.starbase.sysu.edu.cn/), and circbank (http://www.circbank.cn/), were independently used to analyze MRE-based circRNA/microRNA complementation.

### Luciferase reporter assays

To elucidate the circRNA-miRNA interaction, an hsa-circ-0046367 sequence containing the putative target sites for miR-33a was synthesized and cloned into the pMIR-REPORT™ reporter vector supplied by Shanghai GeneChem (Shanghai, China) downstream of firefly luciferase (circFASN-WT). Mutant hsa-circ-0046367 (circFASN-MU) was also generated with the deletion of the complementary sites. After cotransfection of the reporter vector and miR-33a mimics or negative control in 293T cells, firefly luciferase activity was measured using a dual-luciferase assay kit (Promega, Madison, USA) against that of Renilla luciferase. Each assay was repeated for 5 independent experiments.

### Triglyceride and cholesterol assays

For triglyceride and cholesterol measurements, liver tissue or cells were suspended and homogenized in 1 ml of 5% NP-40/ddH2O solution. The amounts of triglycerides and cholesterol were measured using a triglyceride or cholesterol quantification kit supplied by Nanjing Jiancheng Bioengineering Institute (Nanjing, China).

### Oil red O staining

The treated cells were removed, washed twice with PBS, fixed with 4% formaldehyde at room temperature, and washed three times with PBS. The tissues were prepared as frozen sections. To stain the lipids in adipocytes, the cells or sections were treated with filtered Oil red O solution for 1 h at room temperature and washed twice with PBS. Red-stained lipid droplets were observed under a microscope. ImageJ V.1.51 was used to measure the area of a single cell and of lipid droplets in the cell. The ratio of the two values was used to evaluate the lipid content in cells.

### Quantitative real-time PCR

Total RNA was extracted using TRIzol reagent (Invitrogen). RNA concentration was measured by Nanodrop (Thermo Scientific™, USA), and each paired sample was adjusted to the same concentration. For reverse transcription (RT), we used an RT reagent kit purchased from GENESEED (Guangzhou, China). Real-time PCR was subsequently performed using SYBR Premix supplied by GENESEED (Guangzhou, China) on the Applied Biosystems 7500 Real-Time PCR Detection Systems (Bio-Rad, CA, USA). The following primer sequences were used:

Hsa-mir-33a-F: 5′-TATTCCGAGTGCATTGTAGTTGC-3′

Hsa-mir-33a-R: 5′-TATGGTTTTGACGACTGTGTGAT-3′

Hsa-mir-33b-F: 5′-TGCATTGCTGTTGCATTG-3′

Hsa-mir-33b-R: 5′-GAACATGTCTGCGTATCTC-3′

Mmu-mir-33-F: 5′- TGCATTGTAGTTGCATTGCA-3′

Mmu-mir-33-R: 5′-GAACATGTCTGCGTATCTC-3′

Csl-mir-39-F: 5′-ATATCATCTCACCGGGTGTAAATC-3′

Csl-mir-39-R: 5′-TATGGTTTTGACGACTGTGTGAT-3′

Hsa-circ-0046367-F: 5′-CTCGCTTCGGCAGCACA-3′

Hsa-circ-0046367-R: 5′-AACGCTTCACGAATTTGCGT-3′

Mmu-circ-0002842-F: 5′-CACAGTGCTCAAAGGACATGCC-3′

Mmu-circ-0002842-R: 5′-CACCAGGTGTAGTGCCTTCCTC-3′

GAPDH-F: 5′-GTCTCCTCTGACTTCAACAGCG-3′

GAPDH-R: 5′-ACCACCCTGTTGCTGTAGCCAA-3′

Beta-ACTIN-F: 5′-CACCATTGGCAATGAGCGGTTC-3′

Beta-ACTIN-R: 5′-AGGTCTTTGCGGATGTCCACGT-3′

U6-F: 5′-ATTGGAACGATACAGAGAAGATT-3′

U6-R: 5′-GGAACGCTTCACGAATTTG-3′

The fold change in mRNA expression was calculated using the comparative cycle method (2^−ΔΔCt^).

### CircRNA RNase R treatment

Total RNA (5 µg) was incubated for 15 min at 37 °C with or without 3 U/µg RNase R supplied by GENESEED (Guangzhou, China). The RNA was subsequently purified by phenol–chloroform extraction and then subjected to RT-qPCR.

### Western blotting

Cells were washed in ice-cold PBS, and cellular protein was collected by scraping the cells into 50 µl protein extraction buffer. Western blotting was carried out as described previously.^24^ The following antibodies were used in this study: anti-AKT (Catalog No. 4685, 1:1000, Cell Signaling, Shanghai, China), anti-phospho-AKT (Ser-473) (Catalog No. 4060, 1:1000, Cell Signaling, Shanghai, China), anti-FASN (Catalog No. 3180S, 1:1000, Cell Signaling, Shanghai, China), anti-CPT1A (Catalog No. 12252S, 1:1000, Cell Signaling, Shanghai, China), anti-SREBP1 (Catalog No. ab28481, 1:2000, Abcam, Shanghai, China), anti-SREBP2 (Catalog No. ab30682, Abcam, Shanghai, China), anti-ABCG1 (Catalog No. 13578-1-AP, Proteintech, Wuhan, China), and anti-beta-actin (Catalog No. 60008-1-Ig, Proteintech, Wuhan, China).

### Patients

We enrolled patients who received primary liver transplantation from donations after donor death between November 2017 and May 2018 in our center, and the patients were closely followed up in the outpatient clinic. The inclusion criteria were primary liver transplantation, stable blood concentration of immunosuppressive agents, no dyslipidemia before surgery and home-stay conditions. The exclusion criteria were multiorgan transplantation and incomplete data. The diagnostic criteria of dyslipidemia were as follows: low-density lipoprotein greater than 130 mg/dL, triglycerides greater than 150 mg/dL, and a requirement for lipid-lowering agents. This study was approved by the Ethical Committee of our hospital. The procedures were in accordance with the Regulations on Human Organ Transplant, national legal requirements, and the Helsinki declaration. All organs were donated from citizen resources.

### Statistical analysis

All assays were repeated at least three times. Data are presented as the mean ± standard deviation and were analyzed using GraphPad Prism 5. Differences between groups were determined using Student’s *t*-tests or one-way analysis of variance (ANOVA). Student-Newman-Keuls (SNK) was used as the post hoc analysis following ANOVA. The association between the clinicopathological characteristics of the LT recipients and miR-33 expression or concentration of TAC was assessed using the *χ*2 test. Spearman’s correlation analysis was used to evaluate the correlation between triglycerides and the expression level of miR-33a or the concentration of TAC in blood samples. A *P*-value of <0.05 was considered statistically significant.

## Results

### SREBP2 is essential to TAC-related triglyceride accumulation in vivo and in vitro

**Fig. 1:**
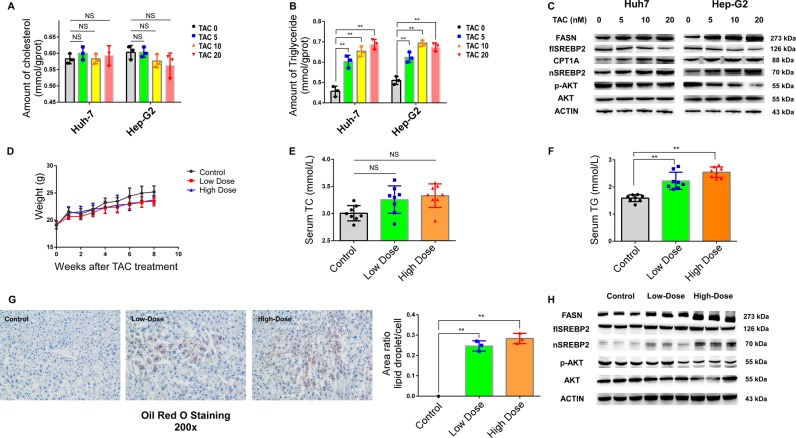
Tacrolimus (TAC) treatment increased the amount of triglycerides in vivo and in vitro. **a**, **b** Triglyceride and cholesterol levels measured by the glycerol lipase oxidase (GPO-PAP) method in Huh7 and Hep-G2 cells treated with different concentrations of TAC. **c** FASN, SREBP2, and AKT protein expression in Huh7 and Hep-G2 cells analyzed by western blotting. **d** Body weight of mice assessed every week in the control (black), low-dose TAC (red), and high-dose TAC (blue) mouse groups (n = 8 per group). **e**, **f** Serum total cholesterol (TC) and triglycerides (TG) assessed 8 weeks after the initial TAC injection. **g** Hepatic triglyceride levels were measured by Oil red O staining. **h** FASN, SREBP2, and AKT protein expression in mouse liver was analyzed by western blotting. Data represent the mean ± SD. *P < 0.05, **P < 0.01, NS, not significant, TAC groups vs. control. The experiments were independently repeated at least three times.

To verify the effect of TAC on lipid metabolism, we created TAC-treated cell and animal models. First, we treated Hep-G2 and Huh7 cells with 5, 10, and 20 ng/ml TAC or DMSO as a control. The intracellular cholesterol quantity was constant across samples, but the relative triglyceride content increased significantly in TAC-treated cells (Fig. 1a, b). TAC can influence lipid metabolism through SREBPs. Fatty acid synthase (FASN), the key lipogenic enzyme catalyzing the terminal steps in the de novo biogenesis of fatty acids, plays an important role in hepatic lipid metabolism and is regulated through SREBPs.^25^ Therefore, we determined the protein levels of SREBPs and FASN and found that the protein levels of SREBP2 and FASN increased in the TAC-treated groups compared with the control groups and were related to the TAC concentration (Fig. 1c).

Next, male C57BL/6 mice were intraperitoneally injected with saline (control), 0.5 mg/kg TAC (low dose), or 2.0 mg/kg TAC (high dose). We did not observe significant differences in body weight or serum cholesterol between the TAC-treated and control groups (Fig. 1d, e). In contrast, the serum triglyceride levels were significantly higher in the TAC-treated groups, particularly the high-dose group (Fig. 1f). Because of the hypertriglyceridemia observed in the TAC-treated groups, we further studied hepatic triglycerides in the mouse livers and found significant accumulation after TAC treatment (Fig. 1g). In addition, the protein contents of SREBP2 and FASN were higher in the TAC-treated groups than in the control groups (Fig. 1h).

These results indicated that TAC treatment could cause the accumulation of triglycerides in the liver through the SREBP pathway.

### Overexpression of miR-33a increases triglyceride (TG) content in vitro through SREBPs and FASN

**Fig. 2:**
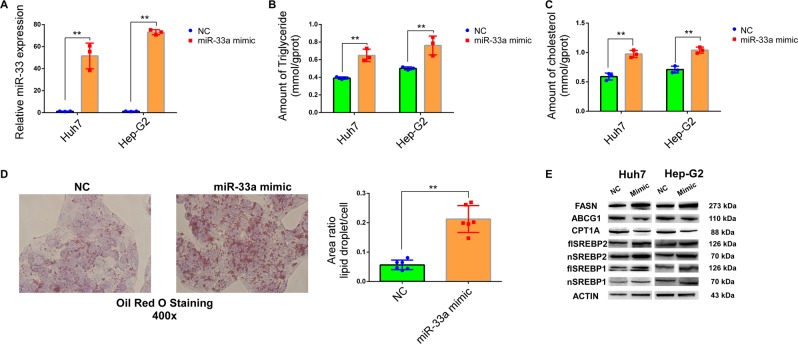
miR-33a is an essential regulator of triglyceride synthesis and metabolism. **a** Transfection efficiency of miR-33a mimic in Huh7 and Hep-G2 cells measured by qPCR. **b**, **c** Triglyceride and total cholesterol levels in Huh7 and Hep-G2 cells increased in the miR-33a mimic groups when measured by the glycerol lipase oxidase (GPO-PAP) method. **d** Triglyceride levels in Hep-G2 cells were measured by Oil red O staining. **e** Protein levels of a few known miR-33a target genes in Huh7 and Hep-G2 cells analyzed by western blotting. Data represent the mean ± SD. *P < 0.05, **P < 0.01, NS, not significant. The experiments were independently repeated at least three times.

**Fig. 3:**
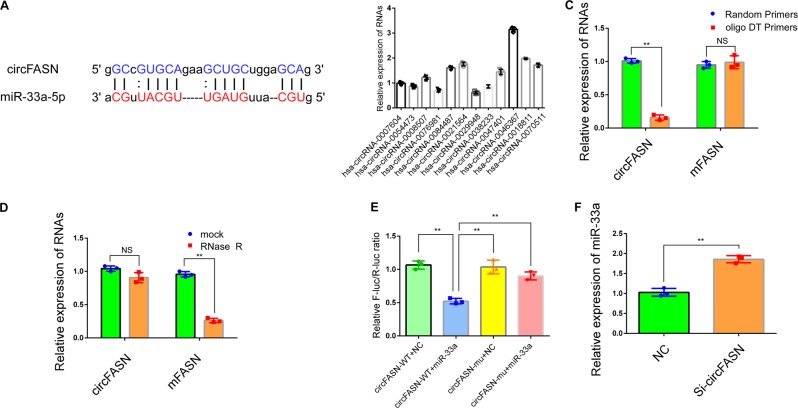
circFASN interacts with miR-33a in the liver. **a** Sequence base pairing between circFASN and miR-33a-5p. The seed region is fully complementary to the sequence of circFASN. **b** Relative expression of predicted miR-33a-targeting circRNAs in Hep-G2 cells detected by qPCR. **c** circFASN could only be amplified by random primers. **d** circFASN is resistant to RNase R treatment. **e** The luciferase reporter assay indicated that circFASN directly interacts with miR-33a-5p at the predicted interaction site in human cells. **f** miR-33a expression increased after the silencing of circFASN in Hep-G2 cells. Data represent the mean ± SD. *P < 0.05, **P < 0.01, NS, not significant. The experiments were independently repeated at least three times.

Since TAC treatment caused triglyceride accumulation via SREBP2 and miR-33a is encoded within SREBP2,^18,19^ we speculated that miR-33a could be a key regulator of TAC-induced triglyceride accumulation. First, we explored the function of miR-33a in triglyceride metabolism. We overexpressed miR-33a by using miR-33a mimics in Hep-G2 and Huh7 cell lines (Fig. 2a). After overexpression, we observed the accumulation of intracellular triglycerides and cholesterol in the mimic groups compared with the control groups (Fig. 2b, c). The accumulation of triglycerides was further verified by using Oil red O staining (Fig. 3d). In miR-33a-mimic-treated cell lines, we observed an increase in SREBP1, SREBP2, and FASN expression (Fig. 2e). Therefore, we concluded that miR-33a likely influenced the amount of TG and TC in vitro through SREBPs and FASN.

### CircFASN can “sponge” miR-33a

The target genes of miR-33a involved in lipid metabolism have been widely reported (Table 1); however, miR-33a-targeting circRNAs have rarely been studied in the liver (Table 2). Considering that circRNAs “sponge” miRNAs to regulate miR target genes posttranscriptionally, we speculated that circRNAs likely regulate lipid metabolism-related genes by interacting with miR-33a.^26,27^ To identify the upstream circRNAs of miR-33a, several circRNAs predicted to target miR-33a were identified based on base complementarity. According to the algorithms of circBase, starBase, and circbank and further screening, we identified 12 miR-33a-targeting circRNAs that might play a role in lipid metabolism. We then analyzed the complementary binding and abundance in cells and selected circFASN for further research (Fig. 3a, b).

**Table 1:** Target genes of miR-33a and their associated pathways.

miRNA	Target gene	Pathway
miR-33a	ABCA1	HDL biogenesis and cholesterol efflux
	ABCG1	HDL biogenesis and cholesterol efflux
	NCP1	
	CROT	Fatty acid biosynthesis
	CPT1a	Fatty acid oxidation
	HADHB	
	CYP7A1	Bile acid synthesis and secretion
	ABCB11	
	ATP8B1	
	IRS2, G6PC	Insulin signaling
	PCK1	Glucose metabolism
	PDK4	Mitochondrial biogenesis
	SLC25A25	
	PGC-1a	
	AMPK	Macrophage polarization

**Table 2:** miR-33a-targeting circRNAs.

circRNA name	Gene Name
hsa_circRNA_0007604	CDC42
hsa_circRNA_0054473	MSH6
hsa_circRNA_0008507	GBA3
hsa_circRNA_0076981	EEF1A1
hsa_circRNA_0084487	UBE2V2
hsa_circRNA_0021564	EIF3M
hsa_circRNA_0029948	PDS5B
hsa_circRNA_0038233	SMG1
hsa_circRNA_0047401	GALNT1
hsa_circRNA_0046367	FASN
hsa_circRNA_0018811	PPP3CA
hsa_circRNA_0070511	PPP3CB

The circular characteristics of circFASN were then identified. Owing to the lack of a poly A tail, circRNAs cannot be reverse transcribed by random primers, and because of their circular form, circRNAs cannot be degraded by RNase R.^28^ As expected, compared to the linear form, circFASN could not be reverse transcribed with oligo DT primers (Fig. 3c). Moreover, circFASN was resistant to RNase R treatment (Fig. 3d). These results indicated that circFASN has a naturally circular RNA form.

We then tested whether circFASN is capable of “sponging” miR-33a in a cellular system. To validate our hypothesis, we performed a dual-luciferase reporter assay in human embryonic kidney 293T (HEK293T) cells. A significant decrease in firefly luciferase activity was observed when cells were cotransfected with SV40-firefly-luciferase-MCS-circFASN-wildtype and miR-33a mimics compared with the control groups cotransfected with scrambled RNA. This suppressive effect could be reversed with mutant circFASN, which disrupted the binding site between the circRNA and miR-33a (Fig. 3e). Moreover, decreased expression of circFASN by using siRNA, which was specific to the back-splicing region of circFASN for silencing, could increase the expression of miR-33a (Fig. 3f). Thus, there is a highly efficient interaction between circFASN and miR-33a via the predicted binding site.

### CircFASN regulates triglyceride metabolism through miR-33a

**Fig. 4:**
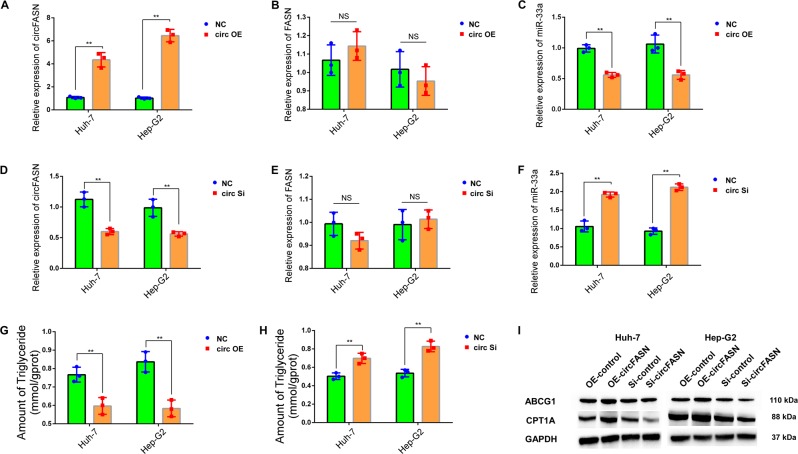
circFASN induced triglyceride accumulation by “sponging” miR-33a. **a** circFASN was successfully overexpressed in Huh7 and Hep-G2 cells. **b** The linear form of FASN was not affected by the circFASN overexpression plasmid. **c** Relative expression of miR-33a, detected by qPCR, was reduced in the circFASN overexpression (OE) groups in Huh7 and Hep-G2 cells. **d** circFASN was silenced in Huh7 and Hep-G2 cells. **e** The linear form of FASN was not affected by silencing circFASN. **f** The relative expression of miR-33a, detected by qPCR, was increased in the circFASN-silenced Huh7 and Hep-G2 cells. **g**, **h** Triglyceride levels in Huh7 and Hep-G2 cells measured by the glycerol lipase oxidase (GPO-PAP) method. **i** Protein levels of ABCG1 and CPT1A in Huh7 and Hep-G2 cells determined by western blotting. Data represent the mean ± SD. *P < 0.05, **P < 0.01, NS, not significant. The experiments were independently repeated at least three times.

To determine whether circFASN regulates lipid metabolism by “sponging” miR-33a, we constructed a circFASN-overexpressing plasmid with a circular frame and the circFASN sequence and found that circFASN could be significantly overexpressed in Hep-G2 and Huh7 cells (Fig. 4a) without affecting the expression of linear FASN (Fig. 4b). After overexpression of circFASN, the expression of miR-33a was reduced as expected (Fig. 4c). We also constructed an siRNA specific to the back-splicing region of circFASN for silencing. After the transfection of circFASN siRNA into Huh7 and Hep-G2 cells, the efficiency of circFASN silencing was measured and confirmed by qPCR (Fig. 4d). The expression of FASN mRNA did not change significantly after the transfection of circFASN siRNA, confirming the specificity of circFASN silencing (Fig. 4e). After the silencing of circFASN, the expression of miR-33a increased (Fig. 4f).

Next, we determined whether circFASN could influence triglyceride levels. After overexpression of circFASN in palmitic acid-treated Huh-7 and Hep-G2 cells, we observed a reduction in cytoplasmic triglyceride in the overexpression groups compared with the negative control groups (Fig. 4g). Furthermore, the amount of cytoplasmic triglycerides was significantly higher in the circFASN-silenced groups than in the normal groups (Fig. 4h). Since we verified that miR-33a is a circFASN target, we tested whether circFASN could regulate miR-33a target genes. In the OE-circFASN and Si-circFASN groups in vitro, we observed the influence of circFASN on the miR-33a target genes ABCG1 and CPT1A (Fig. 4i).

Together, these results suggest that overexpression and silencing of circFASN are feasible in vitro and that circFASN exerts its effects by “sponging” miR-33a.

### TAC can influence the expression of circFASN and miR-33a

**Fig. 5:**
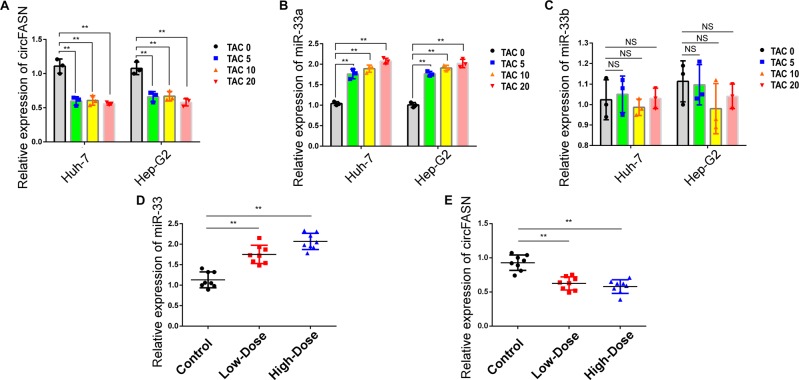
TAC influenced the expression of circFASN and miR-33a. **a** Relative expression of circFASN, detected by qPCR, was reduced in TAC-treated Huh7 and Hep-G2 cells. **b**, **c** Relative expression of miR-33a and miR-33b in TAC-treated Huh-7 and Hep-G2 cells detected by qPCR indicated that TAC only influenced the expression of miR-33a. **d**, **e** The relative expression of mmu-miR-33 and mmu-circFASN was detected in mouse liver by qPCR. Data represent the mean ± SD. *P < 0.05, **P < 0.01, NS, not significant, TAC groups vs. control. The experiments were independently repeated at least three times.

To elucidate the mechanism of TAC-induced triglyceride accumulation, we evaluated the expression of circFASN and miR-33a in TAC-treated models. We treated Huh7 and Hep-G2 cells with TAC and observed a marked TAC-mediated decrease in circFASN expression in all three TAC-treated groups (Fig. 5a). MiR-33a expression was increased as expected (Fig. 5b), and the expression of miR-33b was not significantly different among the TAC-treated and control groups (Fig. 5c).

In the TAC-treated animal model, we observed similar phenomena. We detected the expression of circFASN (mmu_circ_0002842) in the livers of TAC-treated mice. As expected, the expression of circFASN decreased depending on the concentration of TAC (Fig. 5d). Moreover, the expression of miR-33 in the liver increased in both TAC-treated groups (Fig. 5e).

### Overexpression of circFASN can reverse TAC-induced triglyceride accumulation

**Fig. 6:**
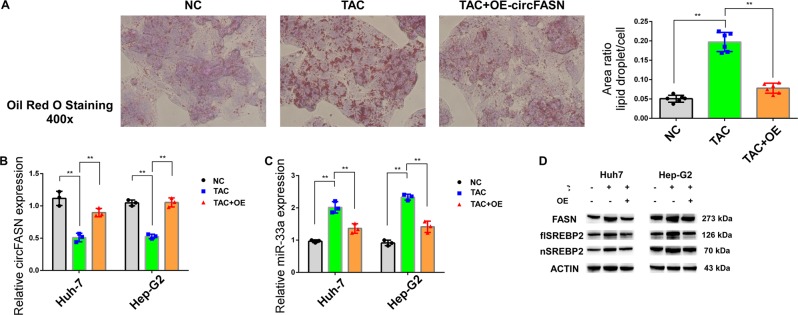
circFASN overexpression blocked TAC-induced triglyceride accumulation. **a** Triglyceride levels in Hep-G2 cells measured by Oil red O staining in the negative control (NC), TAC only -treated, and TAC and circFASN OE plasmid-treated groups. The ratio of the area of lipid droplets to that of a single cell was used to evaluate lipid content in cells. **b**, **c** Relative expression of circFASN and miR-33a in the NC, TAC, and TAC+OE groups detected by qPCR. **d** Protein expression of FASN and SREBP2 in Huh7 and Hep-G2 cells analyzed by western blotting. Data represent the mean ± SD. *P < 0.05, **P < 0.01, NS, not significant, TAC groups vs. the control or OE group. The experiments were independently repeated at least three times.

To further test our hypothesis, we treated cells with TAC- and the circFASN-overexpressing plasmid in a rescue assay. We found clear triglyceride accumulation in TAC-treated groups, which was reversed by circFASN overexpression (Fig. 6a). We found that TAC could decrease the expression of circFASN and increase the expression of miR-33a; however, the reverse trend was observed in Huh-7 and Hep-G2 cells treated with TAC and the circFASN OE plasmid together (Fig. 6b, c). The protein levels of FASN and SREBP2 were upregulated by TAC treatment, and this effect was reversed by overexpression of circFASN (Fig. 6d). Thus, circFASN likely contributes to TAC-associated dyslipidemia after liver transplantation and could be a therapeutic target.

### TAC increases the expression of miR-33a in plasma from LT recipients

**Fig. 7:**
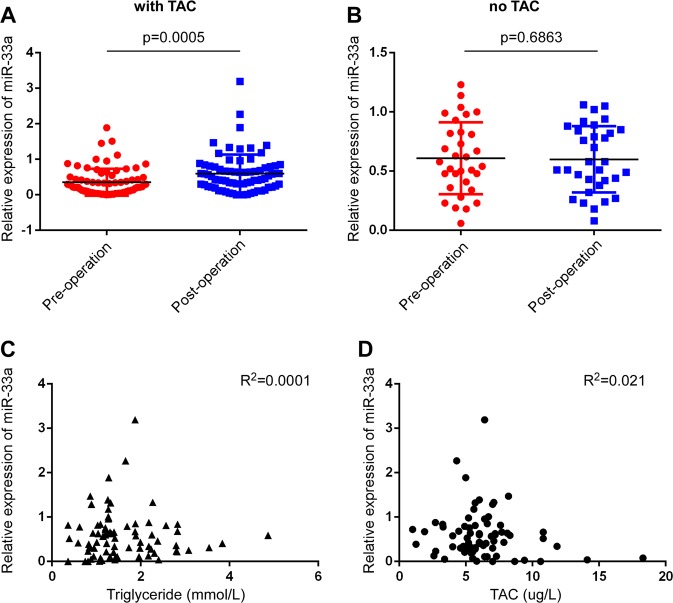
TAC increased the expression of miR-33a in plasma from liver transplant (LT) recipients. **a** The expression of plasma miR-33a increased in LT recipients after surgery. **b** The plasma miR-33a levels showed no significant difference in patients who underwent hepatectomy after surgery. **c**, **d** Plasma miR-33a levels had no correlation with serum triglyceride and blood Fk506 concentrations.

To verify the role of TAC in the regulation of miR-33a and dyslipidemia after LT, we collected plasma from LT recipients before and 3 weeks after surgery. According to our inclusion and exclusion criteria, 73 LT recipients were enrolled and analyzed in this study. We detected the expression level of miR-33a in plasma by quantitative RT-PCR and observed that plasma miR-33a increased after surgery (Fig. 7a). To confirm that TAC was responsible for this increase, we randomly collected samples from 32 patients who underwent hepatectomy in our hospital and did not undergo TAC treatment. As expected, the plasma miR-33a showed no difference pre- and post-surgery (Fig. 7b). This indicated that the use of TAC likely caused an increase in plasma miR-33a. We then analyzed the correlation between the plasma miR-33a level and blood TAC concentration and serum triglyceride. The expression level of plasma miR-33a did not have a significant correlation with blood TAC concentration and serum triglyceride (Fig. 7c, d). We then calculated the fold-change of plasma miR-33a, and based on the receiver operating characteristic (ROC) curve, we calculated a cutoff value for the fold-change to categorize the LT recipients into high and low plasma miR-33a groups. High plasma miR-33a was a risk factor for dyslipidemia after LT (OR 3.742, 95% CI, 1.272–11.012 log-rank, *P* = 0.017). Blood TAC concentration >10 ng/ml is thought to be a risk factor for new-onset diabetes after LT;^29^ however, this was not reflected by our results (Table 3).

**Table 3:** High miR-33a expression in plasma is a risk factor for dyslipidemia after LT.

Characteristics	No. of patients	Dyslipidemia		*P*-value
		Yes, *n* (%)	No, *n* (%)	
miR-33a				
high	41	19 (46.3)	22 (53.7)	*0.012*
low	32	6 (18.8)	26 (81.3)	
TAC concentration at 1 month >10 ng/ml				
Yes	6	2 (33.3)	4 (66.7)	0.666
No	67	23 (34.3)	44 (65.7)	

*miR* microRNA, *TAC* tacrolimus

**Fig. 8:**
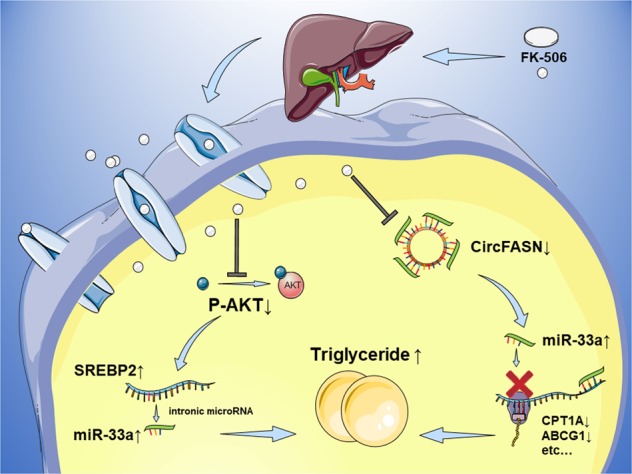
Schematic representation of the circFASN/miR-33a regulatory system in TAC-induced disruption of lipid homeostasis.

## Discussion

There is growing evidence that the circRNA/miRNA/mRNA regulatory system is a novel and important component of gene expression in carcinogenesis and cancer progression.^30–33^ Very little is known about the roles of circRNA/miRNA/mRNA in metabolic processes, particularly hepatocellular lipid metabolism and dyslipidemia. NAFLD and dyslipidemia have a multifaceted impact on liver transplant recipients and organ donors, including the risk of cardiovascular morbidity and mortality.^1^ In the general population, NAFLD and dyslipidemia are associated with obesity, type 2 diabetes mellitus, and MS. Since the liver is a central metabolic organ that plays a key role in lipid synthesis and lipolysis, it is likely important in the development of dyslipidemia, particularly in liver transplant recipients.^7^ The administration of immunosuppressive agents, especially tacrolimus (TAC), also contributes to the etiology of lipid disorders in such patients.^34^ TAC administered after LT may cause lipid metabolism disorders and dyslipidemia. The adverse event profiles of immunosuppressive agents can contribute to the cardiovascular risk in transplant recipients.^35^ However, the pathophysiology and molecular mechanisms underlying these effects remain unclear.

Our previous studies provided the first evidence that TAC induces hepatic glucose dysregulation by modulating miRNAs and their molecular targets.^36^ Considering the close relationship between glucose and lipid metabolism, we hypothesized that TAC-induced disruption of lipid homeostasis could involve microRNA-mediated pathways. MiR-33a is a key regulator of lipid metabolism and is encoded within SREBP2. A study by Rayner et al. aroused considerable interest in miR-33 as a therapeutic target in cardiovascular disease owing to its important role in cholesterol metabolism.^19^ Goedeke et al. showed a significant role for miR-33 in fatty acid metabolism.^37^ We identified a novel role for miR-33a in hepatic lipid metabolism and TAC-induced triglyceride accumulation. Clinically, elevated serum miR-33a after LT is considered a risk factor for TAC-related dyslipidemia. Because of its essential role in hepatic lipid metabolism and potential diagnostic value, miR-33a might serve as a biomarker or therapeutic target for TAC-related metabolic disorders. To avoid the adverse effects of miR-33a inhibition,^38,39^ more precise selection of patients and appropriate modifications, such as nanomaterial wrapping, might be necessary for anti-miR-33a therapy.

Since the first report of a circRNA functioning as a miRNA “sponge”, many physiological and pathological processes have been associated with circRNA-miRNA interactions.^23^ Guo et al. found that circFASN expression is lost during hepatic steatosis, suggesting a circFASN-dependent regulatory system in lipid metabolism.^40^ Bioinformatically, miR-33a was recognized as a target of circFASN based on circBase (http://www.circbase.org/) and miRBase (http://microrna.sanger.ac.uk/) analyses.^41–43^ Experimentally, dual-luciferase reporter assays provided further verification that circFASN acts as a miR-33a “sponge”. Moreover, we confirmed the function of circFASN and miR-33a in TAC-related triglyceride metabolism. We identified miR-33a as a direct target of circFASN and elucidated the function of circFASN/miR-33a related to hepatic triglyceride homeostasis for the first time. Other studies have focused on the target genes of miR-33a, while our study aimed to identify miR-33a-related circRNAs, thus providing new evidence that miR-33a is a key regulator in TAC-induced disruption of lipid homeostasis. Considering the effects on reducing miR-33a and reversing the effect of TAC-induced triglyceride accumulation, circFASN could be used as an adjuvant therapy target together with anti-miR-33a therapy to combat TAC-induced dyslipidemia after liver transplantation.

The pharmacological effects of TAC in different tissues and its impact on glucose and lipid homeostasis via different molecular targets have been reviewed recently.^44^ In the present study, we also extensively studied the effect of TAC administration on the circFASN/miR-33a pathway. Our results provide, to our knowledge, the first evidence that TAC induces hepatic lipid dysregulation by modulating the expression of circFASN and its molecular targets including miR-33. We showed in vivo and in vitro that TAC, at a relatively low concentration, caused downregulation of circFASN accompanied by dysregulation of miR-33a and SREBPs, resulting in hepatocellular lipid metabolism disorders and dyslipidemia. This is similar to patterns observed in recipients of liver transplants. Overall, the adverse effects of TAC on lipid metabolism are quite clear. Therefore, seeking a new immunosuppressant as a proper substitute for TAC would preserve lipid homeostasis and provide a long-term benefit for LT recipients.

Our results indicate that circFASN/miR-33a could serve as diagnostic biomarkers or clinical interference targets for TAC-induced dyslipidemia after liver transplantation. CircFASN/miR-33a normalization by intrahepatic intervention may shed light on potential clinical applications. However, there are some limitations in this study. First, LT is a complicated surgery wherein recipients can experience prolonged anesthesia, operative blood loss, acid-base imbalance, and ischemia-reperfusion injury. Any of these conditions could cause lipid disturbance. However, we only focused on gene polymorphisms of grafts and the impact of TAC after LT. Moreover, the effect of TAC is dose- and time-dependent. We did not explore the effects of a high-dose or long-term TAC treatment. Furthermore, at present miRNA targeting by circRNAs is mostly explained by base complementarity, but it could be more complex. Precise determination of which miRNA binds to a particular circRNA is important for future studies on circRNA function. Finally, the results from murine in vivo and human in vitro experiments need to be verified clinically.

In summary, our findings reveal that the circFASN/miR-33a/SREBP regulatory system represents a novel epigenetic mechanism underlying hepatic steatosis and dyslipidemia after LT (Fig. 8). TAC is a key mediator of this signaling pathway. CircFASN and miR-33a are therefore potential molecular targets for the development of novel therapeutic strategies to reverse TAC-induced metabolic disorders.
